# Proteomic characterization of endogenous substrates of mammalian ubiquitin ligase Hrd1

**DOI:** 10.1186/s13578-018-0245-z

**Published:** 2018-08-22

**Authors:** Yilin Ye, Suk-Hwan Baek, Yihong Ye, Ting Zhang

**Affiliations:** 1 Department of Orthopedics, Peking University First Hospital, Beijing, 100034 China; 2 Department of Biochemistry & Molecular Biology, College of Medicine, Yeungnam University, Gyeongsan, 38541 South Korea; 3 Laboratory of Molecular Biology, National Institute of Diabetes and Digestive and Kidney Diseases, National Institutes of Health, Bethesda, MD 20892 USA; 4 SUSTech Academy for Advanced Interdisciplinary Studies, Southern University of Science and Technology, Shenzhen, 518055 China

**Keywords:** Endoplasmic reticulum-associated protein degradation/ERAD, Protein quality control, OS9, ER stress, ERAD tuning, Lysosome degradation

## Abstract

**Background:**

Endoplasmic reticulum (ER)-associated degradation (ERAD) regulates protein homeostasis in the secretory pathway by targeting misfolded or unassembled proteins for degradation by the proteasome. Hrd1 is a conserved multi-spanning membrane bound ubiquitin ligase required for ubiquitination of many aberrant ER proteins, but few endogenous substrates of Hrd1 have been identified to date.

**Methods:**

Using a SILAC-based quantitative proteomic approach combined with CRISPR-mediated gene silencing, we searched for endogenous physiological substrates of Hrd1. We used RNA microarray, immunoblotting, cycloheximide chase combined with chemical genetics to define the role of Hrd1 in regulating the stability of endogenous ERAD substrates.

**Results:**

We identified 58 proteins whose levels are consistently upregulated in Hrd1 null HEK293 cells. Many of these proteins function in pathways involved in stress adaptation or immune surveillance. We validated OS9, a lectin required for ERAD of glycoproteins as a highly upregulated protein in Hrd1 deficient cells. Moreover, the abundance of OS9 is inversely correlated with Hrd1 level in clinical synovium samples isolated from osteoarthritis and rheumatoid arthritis patients. Intriguingly, immunoblotting detects two OS9 variants, both of which are upregulated when Hrd1 is inactivated. However, only one of these variants is subject to proteasome dependent degradation that requires Hrd1 and the AAA (ATPase associated with diverse cellular activities) ATPase p97. The stability of the other variant on the other hand is influenced by a lysosomal inhibitor.

**Conclusion:**

Hrd1 regulates the stability of proteins involved in ER stress response and immune activation by both proteasome dependent and independent mechanisms.

**Electronic supplementary material:**

The online version of this article (10.1186/s13578-018-0245-z) contains supplementary material, which is available to authorized users.

## Background

The endoplasmic reticulum (ER) is a biosynthetic organelle responsible for the synthesis of ~ 30% of the total eukaryotic proteins. As a result, it is a daunting task to maintain protein homeostasis in this compartment, particularly in secretory cells encountering large protein flux in the secretory pathway. To cope with protein misfolding, cells use the ER-associated protein degradation system (ERAD) [1–3], which eliminates misfolded or unassembled proteins by exporting them from the ER into the cytosol. Subsequently, dislocated proteins are degraded by the ubiquitin proteasome system. This process implicates at least two membrane-bound ubiquitin ligase complexes, which are assembled around Hrd1 and Doa10, respectively [4]. Both Hrd1 and Doa10 are multi-spanning membrane proteins, each bearing a RING (really interesting gene) domain that faces the cytosol [5–7]. These ubiquitin ligases are responsible for ubiquitination and degradation of misfolded ER proteins of difference classes [8]. At least for Hrd1, several lines of evidence suggested that it forms a protein conducting channel in the membrane, allowing misfolded luminal proteins to enter the cytosol for ubiquitination [9–11]. Doa10 and its mammalian homologue March6 likely perform a similar function, but for misfolded proteins bearing a misfolding signal in the cytosol or membrane domains [12]. Once ubiquitinated, ERAD substrates are dislocated into the cytosol by the action of the p97 ATPase complex [1, 13]. Dislocated ERAD substrates are eventually shuttled to the proteasome for degradation. Although Hrd1 is mainly involved in degradation of misfolded ER proteins, certain endogenous proteins with no apparent misfolding issues can also be subject to Hrd1-mediated degradation [6, 14–18]. However, very few endogenous substrates of Hrd1 have been identified to date.

The biologic significance of the ERAD pathway is revealed by the facts that it is highly conserved during the evolution, and that its capacity is often fine-tuned to match the load of misfolded proteins in the ER. Because structurally speaking, misfolded proteins are probably quite similar to proteins in the folding process, over-activation of the ERAD pathway should be avoided to prevent premature elimination of proteins that have not finished the folding process. It is thought that under normal conditions the pathway operates at a relatively low capacity, but under proteotoxic stress conditions such as ER stress, the ERAD activity is elevated [19]. This can be achieved by upregulating the mRNA expression of many ERAD machinery genes. When protein misfolding crisis is over, many ER-associated chaperones or ERAD factors are then degraded by either lysosome or proteasome via a process termed “ERAD tuning” [20]. Among them, HERP is a dedicated ERAD regulator highly induced by ER stress [21], but it is short-lived and constitutively degraded by the proteasome [22, 23]. By contrast, the stress-induced luminal chaperone BiP, which participates in both protein folding and ERAD, is degraded by lysosomes [24]. The degradation of ERAD machinery proteins via these so called ‘ERAD tuning’ processes would conceptually limit the ERAD capacity, allowing it to match the level of misfolded proteins. Currently, whether ERAD machinery proteins are generally regulated by ERAD tuning is unclear.

OS9 is a lectin chaperone that promotes the degradation of misfolded glycoproteins via the Hrd1-dependent ERAD process [25]. It is localized in the ER lumen and serves as a receptor for misfolded proteins bearing mannose-trimmed *N*-glycan [26]. Interaction with OS9 is thought to cause misfolded glycoproteins to exit the protein folding cycle. OS9 is ubiquitously expressed in multicellular organisms, but its expression is highly upregulated in osteosarcoma [27]. By a non-bias proteomic approach, we identified OS9 together with many other ER proteins implicated in stress adaptation and immune surveillance as candidate substrates of Hrd1. We demonstrated that the stability of two OS9 isoforms can be differentially regulated by different disposing mechanisms, but both required Hrd1. The study establishes Hrd1 as a central regulator of ERAD tuning.

## Results

### Identifying endogenous candidate substrates of Hrd1

**Fig. 1:**
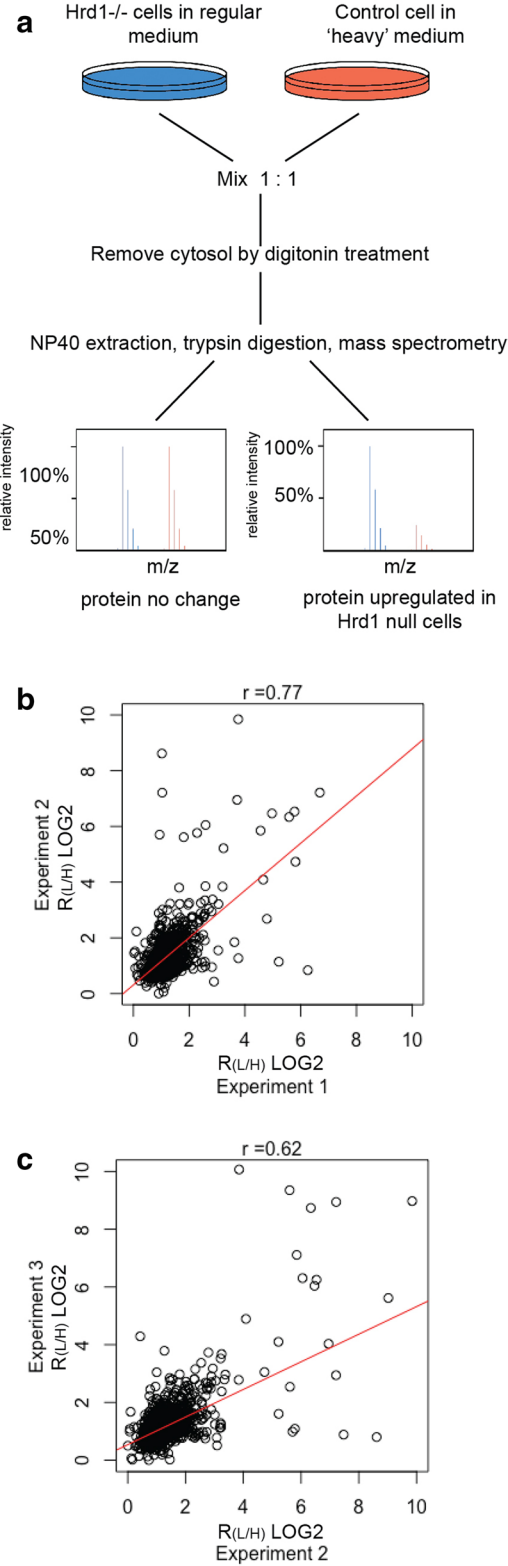
SILAC-based mass spectrometry analyses of Hrd1 deficient and control HEK293 cells. **a** Diagram of the experimental procedure. **b**, **c** Scatter plots demonstrate the reproducibility of three independent experiments. R_L/H_, ratio of light to heavy amino acid

To identify endogenous substrates of Hrd1, we used Hrd1 deficiency HEK293 cells previously generated in our laboratory [28]. Since Hrd1 is a ubiquitin ligase that downregulates proteins via proteasomal degradation, we expected that inactivation of Hrd1 should lead to accumulation of substrates, as demonstrated for misfolded model ERAD substrates [29]. We therefore performed stable isotope labeling with amino acids (SILAC)-based quantitative mass spectrometry analyses (Fig. 1a). To this end, we labeled control wild type (WT) cells with ‘heavy’ amino acids and Hrd1 deficient cells with ‘light’ amino acids. We then mixed equal number of the two cell populations and prepared whole cell extract for analyses by mass spectrometry. From three independent experiments, we identified 2557, 2657 and 3292 proteins, respectively. Among them, many proteins consistently showed an increased ratio of light to heavy amino acids (R_L/H_), suggesting accumulation in the absence of Hrd1 (Fig. 1b, c). Particularly, we identified with high confidence 58 proteins whose R_L/H_ value was increased by at least 2-fold in the absence of Hrd1 (p-value < 0.05) (Additional file 1: Table S1). We reasoned that the stability of these proteins might be regulated by Hrd1-mediated ubiquitination and degradation because several previously identified Hrd1 substrates such as MHC class I heavy chains [30] and NFE2L1 [31] were among the list. However, it is possible that some proteins might be indirectly influenced by knockout of Hrd1 because our study also identified 4 proteins that were downregulated in Hrd1 CRISPR null cells (Additional file 1: Table S1).

### Bioinformatics analyses of candidate Hrd1 substrates

**Fig. 2:**
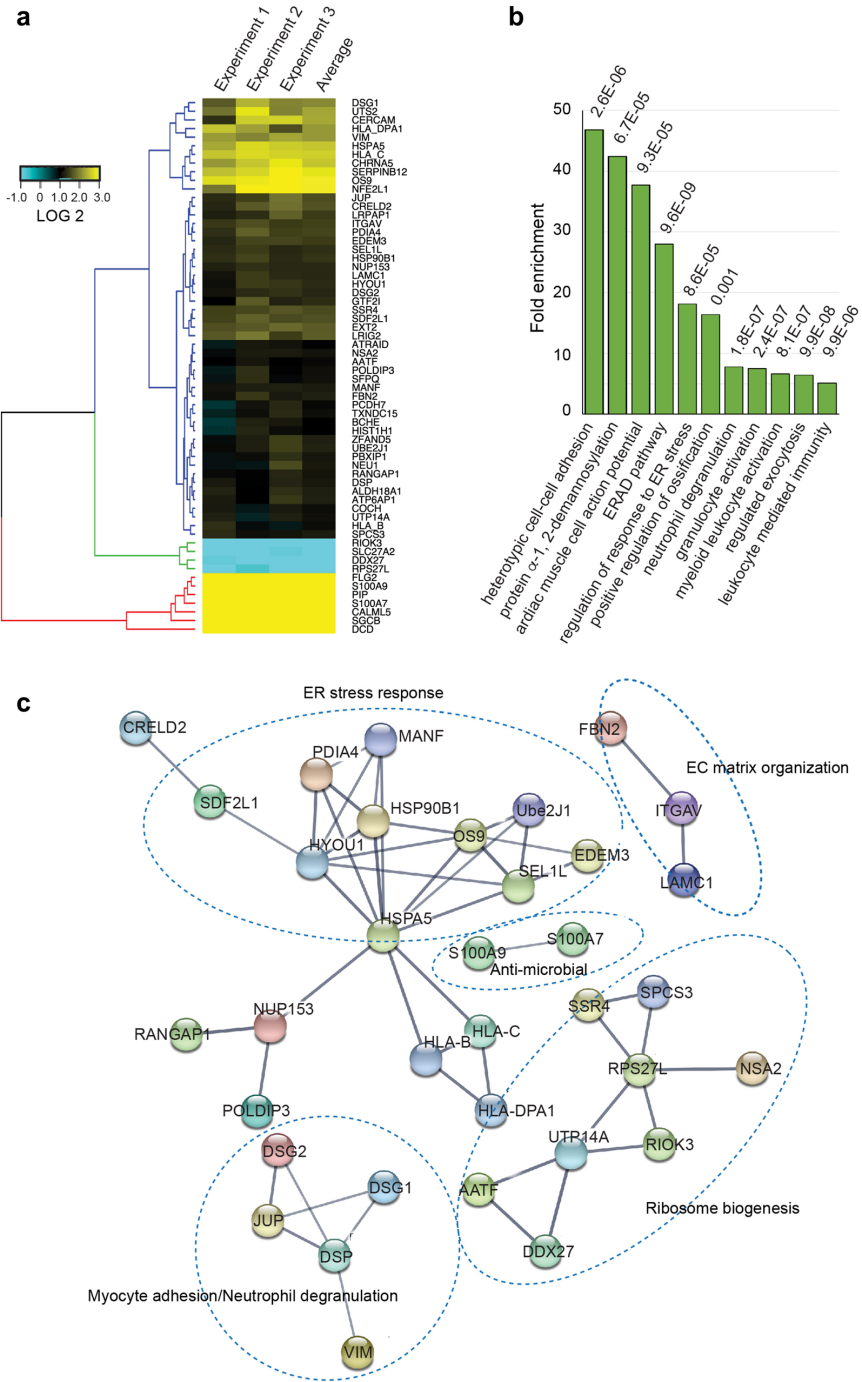
Bioinformatics analysis of candidate Hrd1 substrates. **a** A heat map view of proteins consistently upregulated or downregulated by at least 2-fold in Hrd1 deficient cells. **b** Gene ontology analysis of the identified candidate Hrd1 substrates. **c** Protein interaction networks for the identified proteins as revealed by the STRING database. Proteins in each of the subnetwork were also subject to ontology analysis to identify the underlying molecular pathways

Genes upregulated upon Hrd1 inactivation could be clustered into four major groups depending on the level of upregulation (Fig. 2a). Gene ontology analyses showed that most proteins identified were either membrane proteins or secretory proteins (Data not shown), consistent with Hrd1 being an ER-associated ubiquitin ligase. Moreover, the identified proteins are enriched in several biological processes with the most outstanding ones being ER stress response, leukocyte-mediated immunity and cell-cell adhesion (Fig. 2b). We analyzed the potential protein-protein interactions among the identified proteins using the STRING database. The analysis revealed one large interaction network and several small ones (Fig. 2c). The large interaction network was centered around the heat shock protein HSPA5 (also named BiP), which is an ER-localized Hsp70 family member implicated in ER protein folding and stress response (Fig. 2c). Accordingly, gene ontology analysis showed that this network was involved in ER stress adaptation as it contains many proteins known to play a role in ERAD (e.g. HSP90B1, OS9, SEL1L, EDEM3, UBE2J1, PDIA4). Thus, it seems that a key function of Hrd1 is to restrict the ERAD activity. The small interaction networks include extracellular matrix organization, ribosome biogenesis, myocyte adhesion, and anti-microbial response (Fig. 2c).

### Proteins upregulated in Hrd1 deficient cells are controlled mostly by post-transcriptional mechanisms

**Fig. 3:**
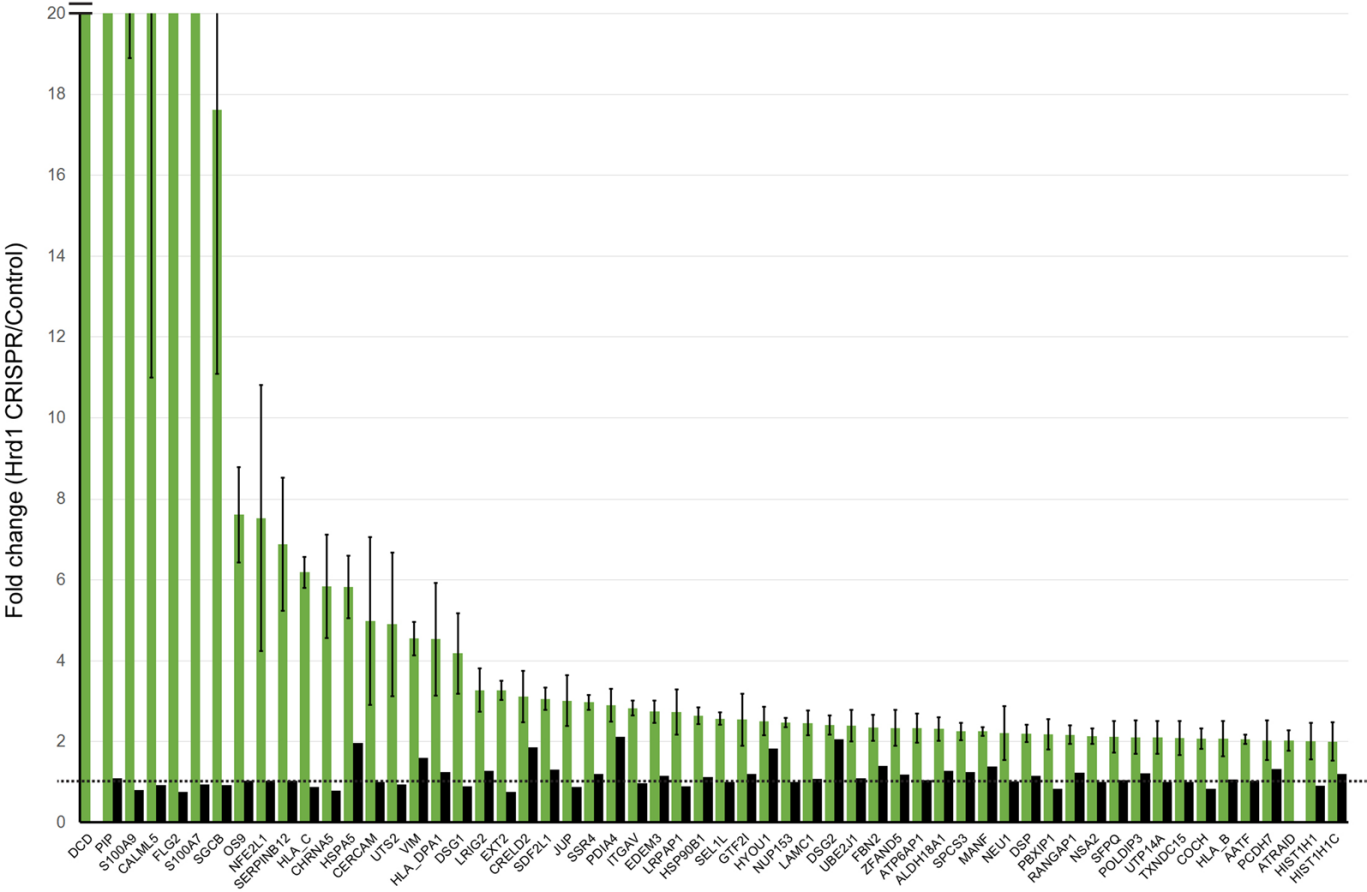
A comparison of the SILAC and microarray studies. The green bars show enrichment (Fold change) for the indicated proteins in Hrd1 deficient cells as determined by 3 independent experiments (error bars, sem, n = 3). The black bars show the corresponding ratio of mRNA between Hrd1 knockout and control cells. Note that most mRNA levels are not changed by Hrd1 depletion

To see whether the identified genes are regulated by Hrd1 post-transcriptionally, we performed microarray analysis using RNA extracted from control and Hrd1 null cells. We performed two independent experiments, which revealed a list of genes whose expression was altered by Hrd1 inactivation (Data not shown). However, a comparison of the proteomic and microarray studies showed that for most proteins accumulated in Hrd1 deficient cells, no corresponding increase in their mRNA level was observed (Fig. 3). This result suggested that the level of these proteins is regulated by Hrd1 via a post-transcriptional mechanism.

### OS9 is upregulated in Hrd1 deficient cells

Many proteins identified are ERAD regulators. Among them, SEL1L, Ube2J, HSPA5, and OS9 are known to interact functionally with Hrd1 [32]. These proteins are likely regulated directly by Hrd1. We decided to use OS9 to validate the ERAD tuning activity of Hrd1 because among the cohort of the ERAD proteins identified, OS9 showed the highest level of up-regulation in Hrd1 null cells, and because commercial OS9 antibodies were able to detect endogenous OS9 (see below). Moreover, upregulation of OS9 in Hrd1 knockout MEF cells was also reported recently [16].

**Fig. 4:**
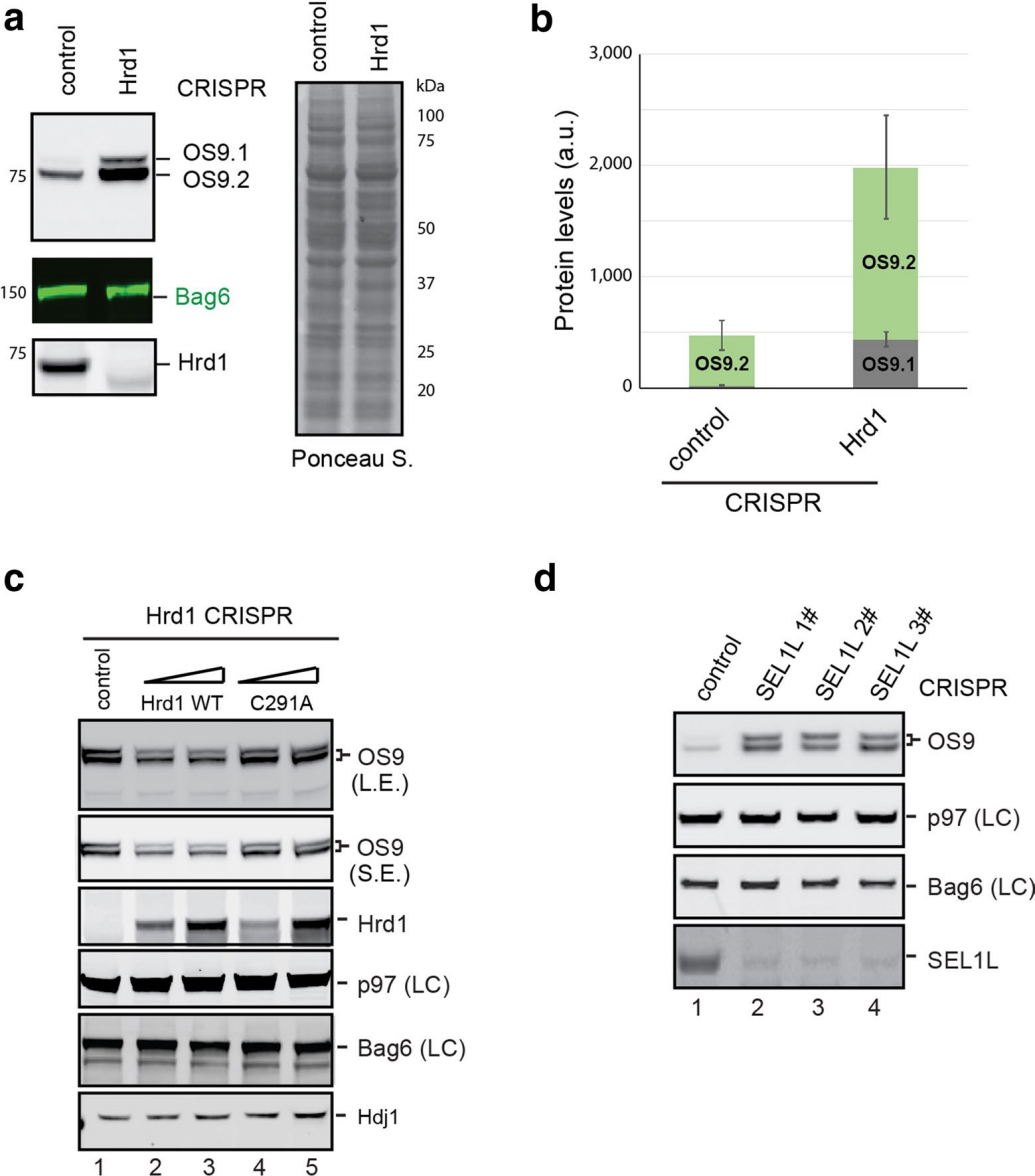
Accumulation of OS9 in Hrd1 and SEL1L knockout cells. **a**, **b** The level of endogenous OS9 was determined by immunoblotting using cell extracts from control or Hrd1 CRISPR null cells. The graph in **b** shows quantification from 3 independent experiments (error bars, sem, n = 3). **c** Overexpression of wild type Hrd1 reduces the level of OS9 in Hrd1 null cells. Where indicated, wild type (WT) or the C291A Hrd1 mutant were transfected into Hrd1 knockout cells at two different doses. Cell extracts were analyzed by immunoblotting. LE, long exposure; SE, short exposure; LC, loading control. **d** Cell extracts from control and three clones of SEL1L CRISPR null HEK293 cells were analyzed by immunoblotting with antibodies against the indicated proteins

We first used immunoblotting to confirm that OS9 is upregulated in Hrd1 deficient cells. Intriguingly, immunoblotting using an OS9 antibody detected two OS9 products, a major band of ~ 75 kD and a weak band slightly above the major one (Fig. 4a). Both these species were OS9 protein because they were not detected in OS9 knockout cells (Data not shown) and because both proteins were significantly upregulated in Hrd1 null cells when compared to control cells (Fig. 4a, b). The effect was specific as cytosolic chaperones such as Bag6 and p97 were not affected by inactivation of Hrd1 (Fig. 4a, c). This phenotype was not due to off-target effect of CRISPR-mediated gene silencing because re-expression of Hrd1 in Hrd1 null cells could reduce the level of both OS9.1 and OS9.2 (Fig. 4c). By contrast, re-expression of a catalytically inactive Hrd1 mutant (C291A) had no effect [33]. Thus, Hrd1 appears to downregulate both OS9 variants in HEK293 cells in a manner dependent on its catalytic activity. For simplicity, we hereafter designated these two OS9 variants as OS9.1 and OS9.2, respectively. Quantification of multiple independent experiments showed that the level of OS9.1 was increased in Hrd1 CRISPR cells by more than 10-fold, whereas OS9.2 was only increased by ~ 3-fold (Fig. 4b). Because SEL1L is a functional partner of Hrd1, we also tested whether the level of OS9 proteins was also up-regulated by SEL1L. Likewise, we used the CRISPR technology to generate SEL1L null cells. Immunoblotting analysis demonstrated that knockout of SEL1L similarly increased the expression of both OS9.1 and OS9.2 (Fig. 4d), suggesting that OS9 is subject to degradation by the Hrd1-SEL1Lcomplex.

### The stability of OS9.1 and OS9.2 are differentially regulated

**Fig. 5:**
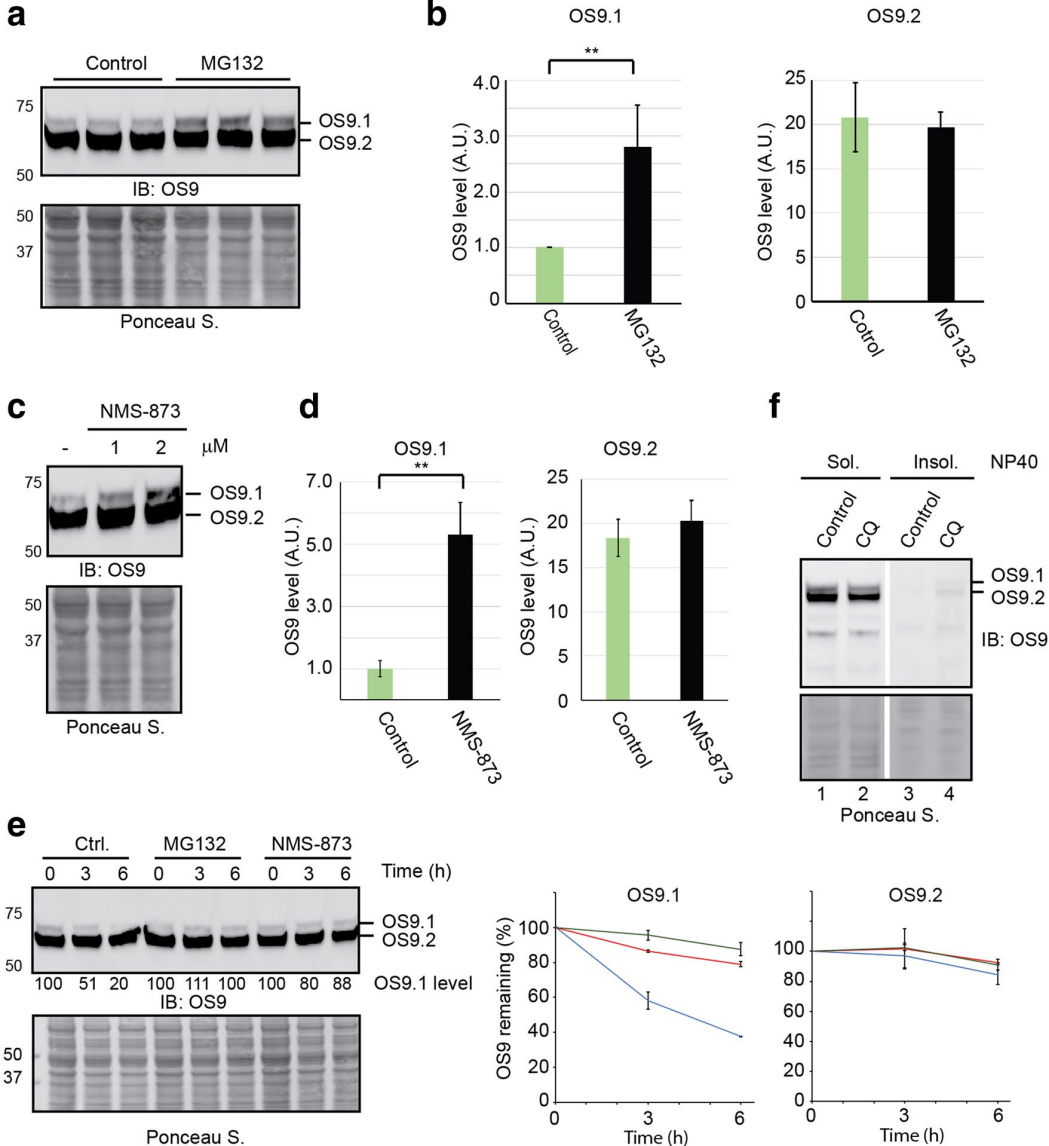
The stability of OS9.1 and OS9.2 are differentially regulated. **a**, **b** Proteasome inhibition increases OS9.1 but not OS9.2. Lysates from cells treated with DMSO (Control) or MG132 (20 µm, 6 h) in triplicates were analyzed by immunoblotting (**a**). The graphs in **b** show the quantification (error bar, sem, n = 3). **p-value < 0.01. **c**, **d** Inhibition of p97 stabilizes OS9.1 but not OS9.2. Cells were treated with the indicated concentration of NMS-873 for 6 h followed by immunoblotting analysis. The graphs in **d** show quantification of 3 treatment experiments using 2 µm NMS-873. **e** Cycloheximide chase analysis of OS9 degradation in cells treated with MG132 and NMS-873. The graphs show quantification of 3 independent experiments. Error bars, sem, n = 3. **f** Chloroquine treatment stabilizes a fraction of OS9 in NP40 insoluble fraction. Immunoblotting analysis of NP40-soluble and insoluble fractions of cells treated with chloroquine (100 µM 16 h)

As the ubiquitin proteasome system is the major protein degradation machinery in eukaryotic cells, we tested whether OS9 variants were substrates of the proteasome. We treated HEK293 cells with the proteasome inhibitor MG132 and analyzed the level of OS9 in whole cell extracts by immunoblotting. Interestingly, the level of OS9.1 was consistently elevated in cells treated with MG132 (~ 3-fold after 6 h treatment). By contrast, OS9.2 was not significantly affected by MG132 treatment (Fig. 5a, b). Thus, OS9.1 is subject to degradation by the proteasome.

Given that OS9 is an ER luminal chaperone whereas the proteasome is localized to the cytosol, degradation of OS9.1 likely requires retrotranslocation of OS9.1 into the cytosol by p97. We therefore also tested whether inhibition of p97 could result in a similar accumulation of OS9.1 [34]. Indeed, the level of OS9.1 but not OS9.2 was increased by ~ 5-fold in cells exposed to the specific p97 inhibitor NMS-873 compared to untreated control cells (Fig. 5c, d). These data suggested that OS9.1, but not OS9.2 was degraded via a mechanism dependent on p97 and the proteasome. This notion was indeed confirmed by cycloheximide chase experiments, which showed that OS9.1 was unstable with a half-life of ~ 3 h, and treatment with either NMS-873 or MG132 almost completely stabilized it (Fig. 5e). By contrast, OS9.2 was only slowly degraded, and its stability was not affected by either MG132 or NMS-873.

Since it is generally thought that the proteasome degrades proteins at a much faster rate than the lysosomes, we reasoned that the stability of OS9.2 might be regulated by lysosomes. We treated cells with a lysosomal inhibitor, chloroquine. Although we did not detect any accumulation of OS9.2 in cell extracts solubilized by the detergent NP40 following chloroquine treatment, we detected a small amount of OS9.2 accumulated in a NP40 insoluble fraction in chloroquine-treated cells (Fig. 5f). This result suggested that a small fraction of OS9.2 is transported from the ER to lysosomes for degradation.

### Hrd1 regulates the stability of OS9.1 and OS9.2

**Fig. 6:**
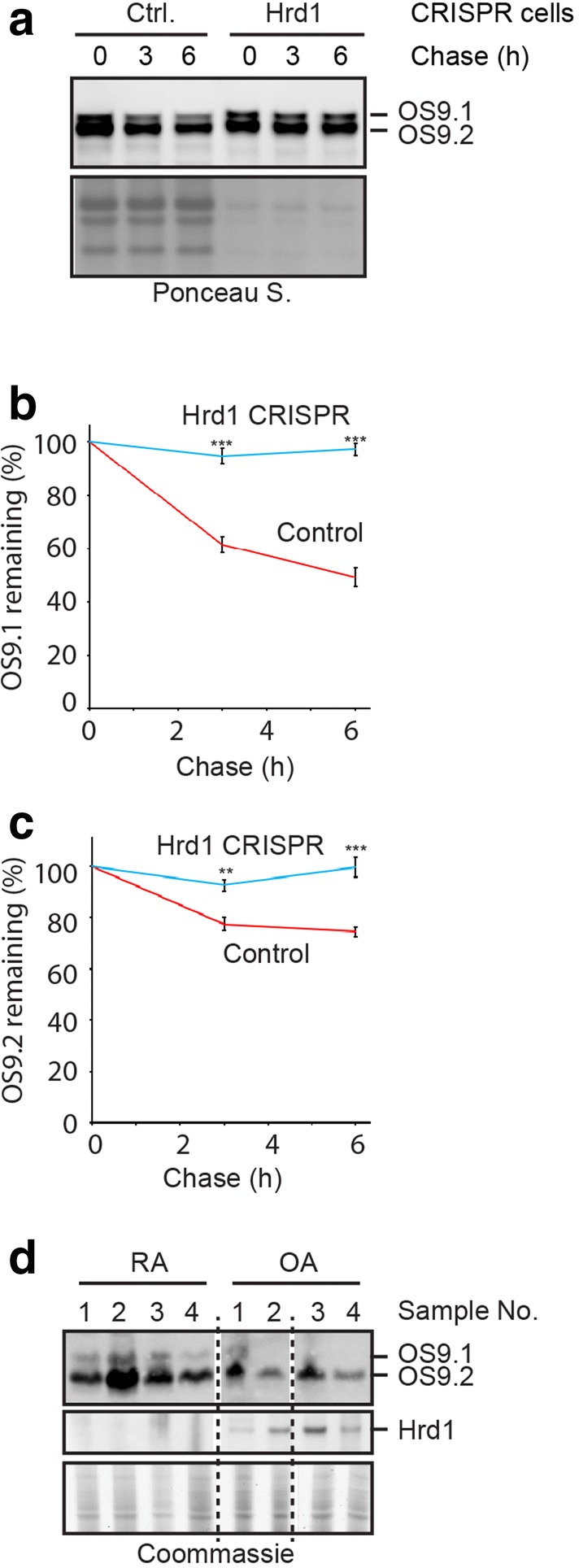
Hrd1 regulates the stability of both OS9.1 and OS9.2. **a**–**c** Cycloheximide chase analysis of OS9 degradation in control or Hrd1 null CRISPR cells. Note that the lysate samples from Hrd1 CRISPR cells were loaded at 20% of the control samples. The graphs in **b**, **c** show quantification of 4 independent experiments. Error bars, sem, n = 4. **p-value < 0.01, ***p-value < 0.005. **d** Synovia isolated from rheumatoid arthritis (RA) or osteoarthritis (OA) were analyzed by immunoblotting

Since inactivation of Hrd1 increased the level of both OS9.1 and OS9.2, it seemed that Hrd1 was capable of regulating both proteasome- and lysosome-mediated degradation of OS9. To test this idea, we performed cycloheximide chase using control or Hrd1 CRISPR cells. Immunoblotting analyses showed that OS9.1 was dramatically stabilized in Hrd1 CRISPR cells. Likewise, the degradation of OS9.2, albeit slow, was also inhibited in Hrd1 null cells (Fig. 6a). We concluded that Hrd1 is required for both proteasome-dependent and -independent degradation of OS9.

Lastly, we wished to test whether the regulation of endogenous OS9 by Hrd1 occurs in vivo. As previous studies have implicated Hrd1 in synovial cell proliferation in rheumatoid arthritis [35], we analyzed the OS9 and Hrd1 protein levels in synovium isolated from patients suffering from either osteoarthritis or rheumatoid arthritis by immunoblotting. We found that synovium from rheumatoid arthritis patients in our collection had a lower level of Hrd1 expression than those from osteoarthritis (Fig. 6d). As anticipated from our cell-based studies, both the OS9.1 and OS9.2 levels in synovium from rheumatoid arthritis were higher than that in osteoarthritis synovium. Although the sample size was relatively small and variables such as patient age and sex could have additional impact on OS9 stability, our data are consistent with the results from cell-based studies, suggesting that Hrd1-dependent regulation of OS9 and other proteins identified by our mass spectrometry studies could play a critical role in synovial cell growth.

## Discussion

### Role of Hrd1 in ‘ERAD tuning’

Hrd1 is part of a multi-protein membrane complex serving a critical role in ERAD, a mechanism that eliminates misfolded proteins from the secretory system. Although its function was best characterized in the context of protein quality control, recent studies have suggested that many endogenously expressed proteins with no apparent folding problem can be regulated by Hrd1-mediated degradation [6, 14–16]. To date, only one study attempted to identify endogenous Hrd1 substrates using a proteomics-based approach [18]. However, the study used Hrd1 knockdown HeLa cells-overexpressing His-tagged ubiquitin, which could alter the stability of many proteins. Indeed, many of the recently confirmed Hrd1 substrates were not among the candidate substrates identified in this study.

In our study, we combined quantitative mass spectrometry with CRISPR-mediated gene silencing to identify endogenous Hrd1 substrates. Our study has revealed a list of proteins whose abundance is altered in Hrd1 deficient cells. As expected from the documented role of Hrd1 in protein degradation, most identified proteins show an accumulation in Hrd1 knockout cells. We show that this is mostly regulated at a post-transcriptional level (Fig. 3). Our study is consistent with a previous report showing that knockout of SEL1L only triggers mild ER stress in mice despite the well documented role of SEL1L and Hrd1 in ER homeostasis regulation [36]. This is presumably caused by cell adaptation during prolonged gene inactivation.

Proteins upregulated upon Hrd1 deletion are enriched in several pathways, ERAD in particular. We validate Hrd1-dependent regulation of OS9, a key ERAD component in this study, which suggests that many ERAD machinery proteins may be degraded via a Hrd1-dependent mechanism. The degradation of OS9.1 is dependent on p97 and the proteasome, suggesting the possibility that ERAD regulators are co-degraded with misfolded proteins. Downregulation of OS9 and other ERAD regulator by Hrd1-dependent mechanisms probably constitutes a major ERAD tuning mechanism that restricts ERAD capacity to a relatively low level until needed.

Intriguingly, we discovered that a fraction of OS9.2 is subject to degradation by a proteasome independent mechanism. Since it was reported that under stress conditions, misfolded proteins may be diverted to lysosome for degradation [37], it is possible that OS9.2 piggy-bags on these substrates to reach the lysosome. This hypothesis is consistent with the observation that inhibition of lysosomal degradation causes the accumulation of a fraction of OS9.2 in a detergent insoluble fraction. Whether the degradation of OS9.2 by lysosome depends on the canonical ER-Golgi-lysosome trafficking route or the recently discovered ER-autophagy pathway remains to be elucidated.

It is worth noting that the coverage of our analysis is limited by the sensitivity of the mass spectrometry instrument. Although we have uncovered several previously established Hrd1 substrates, our list likely does not cover every Hrd1 substrate given that only ~ 10% of the human proteome was recovered. Thus, more Hrd1 substrates may be identified if improvement could be made to increase the sensitivity of the assay.

### Hrd1 and rheumatoid arthritis

Although yeast Hrd1 was initially discovered by genetic screen to search for ERAD regulators, the mammalian Hrd1 homolog was first characterized as a highly expressed protein in rheumatoid synovial cells and was named synoviolin [35]. In mice overexpressing Hrd1, ~ 30% animals develop spontaneous arthropathy, whereas downregulation of Hrd1 protected mice from collagen-induced arthritis [35]. The precise role of Hrd1 in rheumatoid arthritis is unclear. One study suggests that Hrd1 might promote the growth of synovial cells either by stimulating their proliferation or inhibiting apoptosis. Although our study cannot distinguish between these models, our results suggest that abnormal accumulation of endogenous substrates in individuals who have altered Hrd1 expression may be a contributing factor. We reported that synovia from rheumatoid arthritis patients have lower levels of Hrd1 than those from osteoarthritis patients. This conclusion is not consistent with a previous report, which analyzed Hrd1 expression in synovia from two patients of each disease category [35]. Because of the difficulty in obtaining large sample size and in working with human tissues, whether or not Hrd1 expression is altered in rheumatoid arthritis remains to be elucidated. Nevertheless, since our study showed that candidate Hrd1 substrates include many proteins involved in the degranulation process during leukocyte activation, accumulation of these proteins is more consistent with the inflammation phenotype widely observed in rheumatoid but not osteoarthritis joints. The approach developed here, if successfully adapted to rheumatoid synovial cells, may help to clarify Hrd1 substrates directly relevant to rheumatoid arthritis.

## Conclusion

The ubiquitin ligase Hrd1 regulates the stability of endogenous proteins involved in ER stress adaptation and immune surveillance by both proteasome dependent and independent mechanisms. Deregulation of this process may contribute to the abnormal proliferation of synovial cells in rheumatoid arthritis.

## Methods

### Cell lines and reagents

HEK293T cells were purchased from ATCC. Hrd1 and SEL1L CRISPR null and control cells were described previously [28]. Cells were maintained in DMEM medium (Corning cellgro) containing 10% fetal bovine serum and penicillin–streptomycin. OS9, p97, SEL1L antibodies was purchased from Proteintech (10061-1-AP), Fitzgerald (10R-P104A), and Sigma (S3699), respectively. Cycloheximide and chloroquine were purchased from Sigma. MG132 was purchase from Calbiochem, NMS-873 was purchased from Selleckchem (S7285).

To generate SEL1L CRISPR null cells, the following primers corresponding to SEL1L 165-187 and 126-148 were synthesized and annealed. The double-stranded DNAs were cloned into pX330-U6-Chimeric_BB-CBh-hSpCas9 D10A vector [28].

- SEL1L1 CR 1-F 5′-CACCGAGCTTGGCCTCGGCGTCCT
- SEL1L1 CR 1-R 5′-AAACAGGACGCCGAGGCCAAGCTC
- SEL1L1 CR 2-F 5′-CACCGCAGCAGCGTCAGCCCTATC
- SEL1L1 CR 2-R 5′-AAACGATAGGGCTGACGCTGCTGC

### Quantitative mass spectrometry analysis by SILAC

To compare the protein level between control and Hrd1 null cells, we adopted a previously reported SILAC method [38]. In brief, we grew control cells in medium containing heavy amino acids and Hrd1 null cells in regular medium for 1 week. During this period, cells were subcultured two times at 1:10 passages. Cells were then collected and washed with 10 ml phosphate-buffered saline (PBS) and the cell number was counted. Two cell populations were mixed at 1:1 ratio and a total of 4.5 million cells were lysed in a lysis buffer containing 50 mM Tris–HCl pH 7.5, 0.5% NP40, 150 mM sodium chloride, and 5 mM magnesium chloride, 1 mM DTT, and a mixture of protease inhibitors. The lysate was subject to centrifugation at 17,000*g* for 10 min. After determination of protein concentration by the Protein Assay Dye Reagent (BioRad), the protein concentration in cell extract was adjusted to 4.8 mg/ml using the NP40 lysis buffer.

For mass spectrometry analysis, 0.4 ml lysate was mixed with 3.2 ml ice-cold acetone to precipitate proteins. After mixing, we added 0.4 ml 100% trichloroacetic acid (TCA) to the solution. After incubation at − 20 °C for 1 h, precipitated proteins were spun down at 17,000*g* for 10 min. The pellet was washed once with ice-cold acetone. The protein sample was eventually denatured in urea, reduced and alkylated, digested down to tryptic peptides, desalted by reverse phase, separated into six fractions using cation exchange. Data collected using long gradient LC/MS/MS were analyzed by MaxQuant.

To analyze the protein interaction network, the STRING database was used (https://string-db.org/). The gene ontology study was performed using the tool at http://geneontology.org/page/go-enrichment-analysis.

### RNA preparation, array hybridization and quantitative RT-PCR

Total RNA was extracted using TRIzol reagent (Invitrogen) and subsequently purified using an RNeasy MinElute Cleanup kit (QIAGEN). For array hybridization, RNA samples were processed in triplicate and analyzed by the National Institute of Diabetes and Digestive and Kidney Diseases (NIDDK) Genomic Core Facility. Affymetrix gene expression analysis array for the human HG-U133A20 were used (Affymetrix). The microarray signals were analyzed using the Affymetrix RMA algorithm.

### Immunoblotting, protein level measurements and statistical analyses

Immunoblottings were performed using standard protocols. Cells were lysed in a buffer containing 50 mM Tris-HCl pH 7.5, 0.5% NP40, 150 mM sodium chloride, and 5 mM magnesium chloride, 1 mM DTT, and a mixture of protease inhibitors. After centrifugation at 17,000*g* for 5 min, supernatant fractions were saved as NP40 soluble extracts. To obtain NP40 insoluble fraction, the pellet fractions were resuspended in PBS and then mixed with the protein sample buffer. The samples were heated at 95 °C for 20 min prior to SDS-PAGE electrophoresis. Immunoblotting signal was detected by enhanced chemiluminescence method (ECL) (Millipore, WBKLS0100) and recorded by a Fuji LAS-4000 imager. The intensities of protein bands were quantified by ImageGauge v3.0. Alternatively, immunoblotting with fluorescence labeled secondary antibodies was used to quantify abundant proteins. In this case, blots were scanned by a LI-COR Odyssey scanner, and the intensity of protein bands was determined by the Odyssey software. For cycloheximide chase experiments, cells were treated with medium containing 50 µg/ml cycloheximide in the presence or absence of the indicated inhibitors. Cells were then harvested at the time points indicated in the figures for immunoblotting analyses. Synovia from RA or OA patients were obtained during total knee arthroplasty (TKA). Following midline skin incision, the capsule was opened along the medial border of the patella and extended both proximally and distally. Synovial tissue samples were obtained from suprapatellar bursa. For the rheumatoid Arthritis Group, all patients are female with average age of 69.3y. All the patients have typical clinical syndromes of Rheumatoid arthritis including swelling, pain, disfunction of involved knees. The diagnosis was confirmed by the positive laboratory tests including rheumatoid factor (RF) and anti-CCP antibodies preoperatively. For the osteoarthritis Group, all patients are female with average age of 61.8y. All the patients have the typical clinical syndromes of Osteoarthritis: pain, crepitus, disfunction and varus deformity of involved knees. Diagnosis was confirmed by X-ray and negative laboratory tests.

All gels shown are representatives of at least two independent experiments. The n values in the graphs indicate the number of independent experiments. Error bars show mean ± sem, and p values were calculated using paired Student’s t-test.

## Additional file

**Additional file 1: Table S1.** Additional table.
